# Sciatic Nerve Injury Related to Hip Replacement Surgery: Imaging Detection by MR Neurography Despite Susceptibility Artifacts

**DOI:** 10.1371/journal.pone.0089154

**Published:** 2014-02-18

**Authors:** Marcel Wolf, Philipp Bäumer, Maria Pedro, Thomas Dombert, Frank Staub, Sabine Heiland, Martin Bendszus, Mirko Pham

**Affiliations:** 1 Department of Neuroradiology, University of Heidelberg, Heidelberg, Germany; 2 Department of Neurosurgery, University of Ulm, Günzburg, Germany; 3 Center for Peripheral Nerve Surgery, Dossenheim-Heidelberg, Germany; 4 Section of Experimental Radiology, University of Heidelberg, Heidelberg, Germany; Heidelberg University Hospital, Germany

## Abstract

Sciatic nerve palsy related to hip replacement surgery (HRS) is among the most common causes of sciatic neuropathies. The sciatic nerve may be injured by various different periprocedural mechanisms. The precise localization and extension of the nerve lesion, the determination of nerve continuity, lesion severity, and fascicular lesion distribution are essential for assessing the potential of spontaneous recovery and thereby avoiding delayed or inappropriate therapy. Adequate therapy is in many cases limited to conservative management, but in certain cases early surgical exploration and release of the nerve is indicated. Nerve-conduction-studies and electromyography are essential in the diagnosis of nerve injuries. In postsurgical nerve injuries, additional diagnostic imaging is important as well, in particular to detect or rule out direct mechanical compromise. Especially in the presence of metallic implants, commonly applied diagnostic imaging tests generally fail to adequately visualize nervous tissue. MRI has been deemed problematic due to implant-related artifacts after HRS. In this study, we describe for the first time the spectrum of imaging findings of Magnetic Resonance neurography (MRN) employing pulse sequences relatively insensitive to susceptibility artifacts (susceptibility insensitive MRN, siMRN) in a series of 9 patients with HRS procedure related sciatic nerve palsy. We were able to determine the localization and fascicular distribution of the sciatic nerve lesion in all 9 patients, which clearly showed on imaging predominant involvement of the peroneal more than the tibial division of the sciatic nerve. In 2 patients siMRN revealed direct mechanical compromise of the nerve by surgical material, and in one of these cases indication for surgical release of the sciatic nerve was based on siMRN. Thus, in selected cases of HRS related neuropathies, especially when surgical exploration of the nerve is considered, siMRN, with its potential to largely overcome implant related artifacts, is a useful diagnostic addition to nerve-conduction-studies and electromyography.

## Introduction

Hip replacement surgery (HRS) is one of the most frequent orthopedic surgical procedures, with approximately 200,000 total hip replacements, 100,000 partial hip replacements, and 36,000 revision hip replacements annually performed in the United States in 2003, with a strong upward trend in an aging western population. The majority of patients undergoing HRS are sixty-five years of age or older and suffer from at least one or more comorbid disease [1]. Sciatic neuropathy is a rare, but typical complication of HRS. In the literature, its frequency of occurrence ranges from 0.17% to 7.6% [2]–[6].

HRS procedure related sciatic neuropathies rank second after gluteal injection injury as the most frequent cause of traumatically induced iatrogenic sciatic neuropathies [2], [3], [7], [8]. These iatrogenic neuropathies often result in severe and debilitating loss of motor and/or sensory function, are often associated with severe neuropathic pain syndromes and regularly lead to medico-legal claims [9]. Particular surgical risk factors of injury to the sciatic nerve are revision- and posttraumatic- surgery, multimorbidity and female sex [10], [11]. Several potential intraoperative or periprocedural mechanisms of HRS including direct sharp or blunt trauma, compression by surgical material (e.g. clips, wires, sutures, or extrusion of cement) or by intraneural or perineural hematoma, vascular compromise, stretching through excessive lengthening of the leg, or heat of polymerizing cement may injure the sciatic nerve. In many cases the responsible injury mechanism cannot be specifically determined by any diagnostic test [12], [13]. For this reason, it is particularly important to recognize those few patients with direct mechanical compromise, who would benefit most from surgical exploration to release and/or reconstruct the injured nerve segment, and for whom spontaneous satisfactory regeneration would not occur. The predominant injury pattern of clinical symptoms and electrophysiological findings involve the motor distribution of the peroneal division of the sciatic nerve. Therefore clinical symptoms may in certain cases be similar to further distal injuries of the common peroneal nerve. Especially the differentiation of a direct intraoperative injury of the sciatic nerve from a perioperative nervous compression by inadequate positioning, in most cases affecting the common peroneal nerve at its predilection site at the neck of the fibula, is of therapeutic and prognostic importance.

The diagnostic assessment of HRS procedure related sciatic neuropathy includes thorough neurologic physical and electrophysiologic examinations. Conventional radiographs (CR) and/or computed tomography (CT) are performed to evaluate osseus structures, osseus healing, bone and joint positions, and the implant itself [14], [15]. CR/CT are effective in diagnosing fracture, migration or loosening of prosthetic material. Obviously, CR/CT are not capable to adequately depict soft tissue and in particular nervous tissue. High frequency ultrasound has a spatial resolution of up to 400 µm but is limited in its depth of tissue penetration. Therefore deep running peripheral nerves are poorly identified, in particular at proximal body regions.

MRI can be optimized to image the peripheral nervous system, and may then be referred to as MR neurography (MRN). MRN is increasingly being investigated as a potential diagnostic method to determine and localize nerve lesions [16], [17]. So far MRN has never been investigated in HRS procedure related nerve injury, nor have any attempts been reported for the optimization of MRN for sciatic nerve imaging with regard to reducing implant-related susceptibility artifacts in the periprosthetic region.

In this study we used MRN optimized for susceptibility image artifact suppression. Thus, we refer to this method as susceptibility insensitive MRN (siMRN). We show that HRS related sciatic nerve injury may be detected precisely at the fascicular level, i.e. the predominant lesion of the peroneal division of the sciatic nerve was visualized and objectified by measuring T2 signal. Furthermore, we show that patients suffering from direct mechanical compromise after HRS can be identified by siMRN. In these patients, siMRN was crucial to facilitate early surgical intervention to release the constricted nerve and allow nerve regeneration.

## Methods

### Patients and Controls

From December 2009 to February 2012, 9 consecutively referred patients (2 male and 7 female; mean age 61.9 years; age range 49–78 years) with clinically and electrophysiologically suspected sciatic nerve palsy after HRS were prospectively included. 10 healthy volunteers (6 male and 4 female; mean age 52.3 years; age range 50–56 years) served as controls, without any symptoms indicative of peripheral neuropathy, and also without any significant risk factors for polyneuropathy such as diabetes, alcoholism, any infectious or autoimmune disease, peripheral arterial occlusive disease, chronic renal or hepatic disease.

Patient data including age, sex, time since HRS, clinically and electrophysiologically affected nerve, and muscle strength according to the Medical Research Council (MRC) scale of affected target muscles are summarized in Table 1.

**Table 1:** Patient demographics, clinical data, quantitative and qualitative imaging findings.

Patient	Age	Sex	TimesinceHRS(months)	Clinicallyaffectednerve	Muscle strength(MRC) of ipsilateralparetic muscles	Nerve-to-musclesignal intensityratio (SR)	Denervation oftarget musclesat thigh level	Denervation oftarget musclesat calf level	Imagingdiagnosis
1	56	F	4	right sn(peroneallyaccentuated)	0/5 extensors offoot and toes, 4/5flexors of foot	p: 2.3, t: 1.1	none	n.a.	constriction of theperoneal portion ofthe sn by a cerclage
2	78	M	24	left sn(peroneallyaccentuated)	1/5 extensors offoot and toes	p: 1.8, t: 2.2	long headof bfm	atm, edbm, edlm,plm, tibialis ptm,gm	compression of thesn by a small,susceptibility proneforeign body
3	60	F	3	right sn(peroneallydivision or nerve)	2/5 extensors offoot and toes	p: 3.0, t: 2.3	long and shorthead of bfm	atm, edbm, edlm,pbm, plm, medialhead of gm	peroneallyaccentuated,pertrochantericlesion of the sn
4	49	F	2	left sn(peroneallyaccentuated)	0/5 extensors offoot and toes, 4/5flexors of theknee	p: 2.3, t: 1.8	short headof bfm	n.a.	peroneallyaccentuated,pertrochantericlesion of the sn
5	57	F	3	left sn(peroneallyaccentuated)	2/5 extensors offoot and toes, 4/5flexors of theknee	p: 3.5, t: 3.4	short headof bfm, sem	atm, edbm, edlm,pbm, plm, ptm,pom, lateral headof gm	proximal lesion ofthe sn at the ischialtuberosity
6	74	F	2.5	left sn(peroneallyaccentuated)	0/5 flexors of footand toes, 4/5flexors of theknee	sn (peronealand tibialdivision): 4.0	None	atm, edbm,edlm,ptm, pom, medialhead of gm	pertrochantericlesion of the sn
7	55	M	4	right sn(peroneallyaccentuated)	0/5 extensors ofthe foot and toes,4/5 flexors of theknee, flexors ofthe foot and toes	p: 4.1, t: 2.7	None	atm, edbm, edlm,pbm, plm	peroneallyaccentuated,pertrochantericlesion of the sn
8	53	F	12	right sn(peroneallyaccentuated)	1/5 extensors ofthe foot and toes	p: 2.2, t: 1.5	None	atm, edbm,edlm,pbm, plm	peroneallyaccentuated,pertrochantericlesion of the sn
9	75	F	6	left sn(peroneallyaccentuated)	0/5 extensors ofthe foot and toes	p: 2.0, t: 1.5	short headof bfm	atm, edbm, edlm,pbm, plm	peroneallyaccentuated,pertrochantericlesion of the sn

Patient data, including age (in years), sex, time since HRS (in months), clinically and electrophysiologically affected nerve, muscle strength of affected muscles according to the MRC scale, ratio of T2- signal intensity of the peroneal and tibial nerve related to normal appearing musculature (SR), denervation pattern of target muscles at thigh and calf level, and conclusive imaging diagnosis. F: female, M; male, n.a.: not available, SR: nerve-to-muscle T2- signal intensity ratio. **Abbreviations:** atm: anterior tibialis muscle; bfm: biceps femoris muscle; edbm:extensor digitorum brevis muscle; edlm: extensor digitorum longus muscle; gm: gastrocnemius muscle; p: peroneal portion of the sciatic nerve or peroneal nerve; pbm: peroneus brevis muscle; plm: peroneus longus muscle; pom: popliteus muscle; sem: semimembranosus muscle; sn: sciatic nerve; t: tibial portion of the sciatic nerve or tibial nerve; ptm: posterior tibialis muscle.

### Ethics Statement

The study was approved by the institutional review board (University of Heidelberg ethics committee; S-057/2009), and written informed consent was obtained from all participants.

### MR Imaging

All MRN studies were performed on a 3 Tesla MRI scanner (Magnetom TRIO or VERIO, Siemens; Erlangen, Germany). Patients and controls were examined in supine position with standard body matrix coils positioned around hip and upper thigh, and an 8-channel knee coil to examine the distal thigh and proximal calf. Imaging slabs were transversally angulated, perpendicularly to the femoral bone. The following pulse sequences were performed: T2-weighted turbo spin echo (repetition time: 3700 msec; echo time: 56 msec; echo train length: 13; slice thickness: 3,5 mm; number of slices: 35; acquisition matrix: 512/333; field-of-view was individually adjusted to each subject’s girth, ranging from 129×129 to 200×200), T1-weighted turbo spin echo (repetition time: 858 msec; echo time: 11 msec; echo train length: 3; slice thickness: 5 mm; number of slices: 61; acquisition matrix: 448/243; field-of-view was individually adjusted to each subject’s girth, ranging from 160×160 to 200×200). In 7 of 9 cases, in which a proximal lesion of the lumbosacral plexus could not have been ruled out neither by clinical examination nor electrophysiology, visualization of nerve roots and the lumbosacral plexus was performed with a coronally angulated T2 SPACE STIR sequence (repetition time: 3800 msec; echo time: 266 msec; inversion time: 180 msec; echo train length: 249; slice thickness: 1 mm; number of slices: 104; acquisition matrix: 320/316; field-of-view: 305×305). Efforts for reducing metal- related susceptibility artifacts in siMRN included positioning of the patient, choice of pulse sequences, and setting of sequence parameters. As far as possible, the patients were positioned with the long axis of the implant parallel to the static magnetic field (B0). We performed SE sequences, instead of GRE sequences. At the maximal diameter of the implant we used STIR- sequences instead of frequency selective fat- saturation, with low section thickness, a relatively small field-of-view, and a high echo train length. For evaluation of the continuity of the sciatic nerve, T1- weighted SE sequences without fat- saturation were used, being less vulnerable to susceptibility artifacts than the T2- weighted sequence that we used.

### Image Analysis

**Figure 1:**
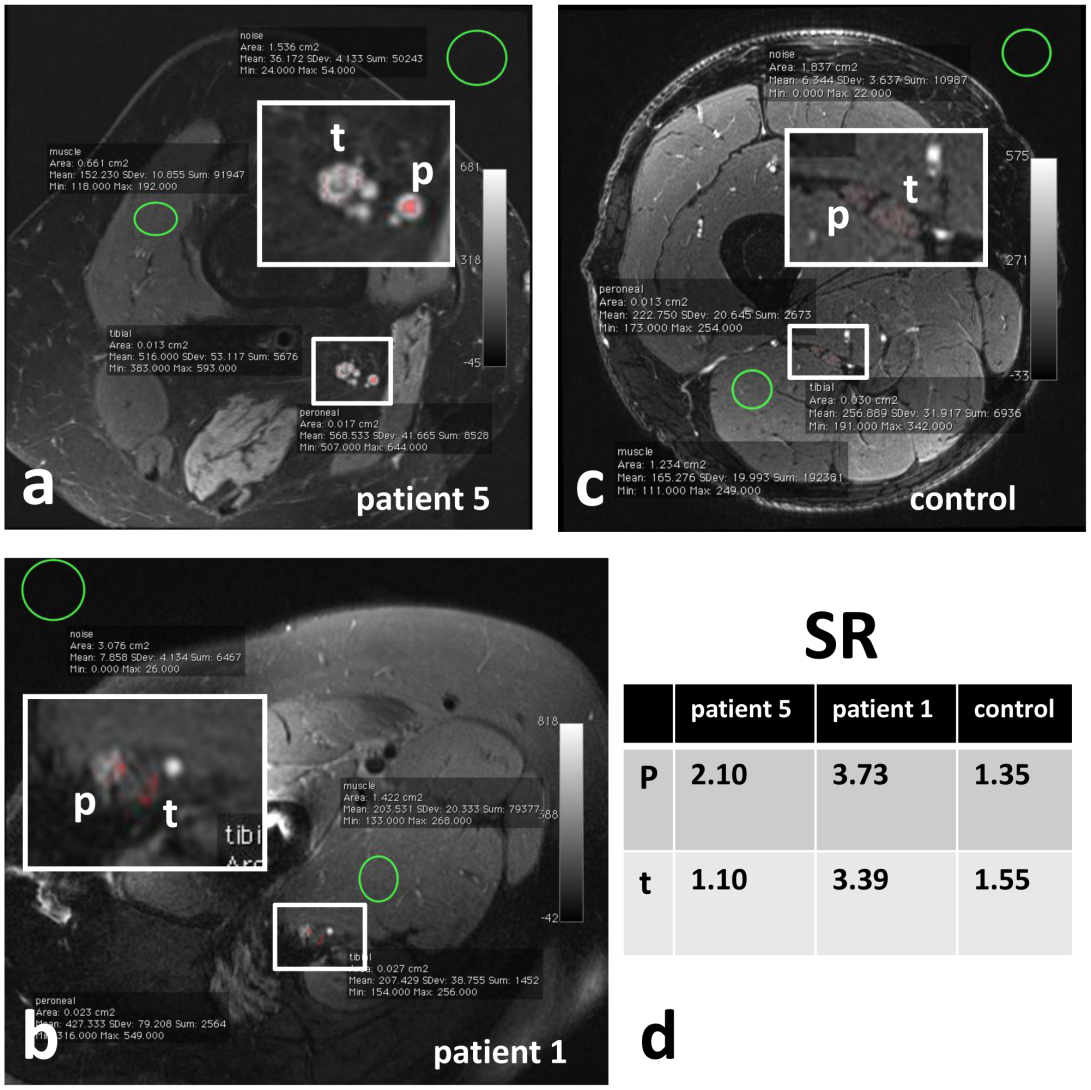
ROI-based measurement of T2-signal intensity of muscle, peroneal and tibial division of the sciatic nerve. siMR neurographic T2-weighted images with fat-saturation (a, b, c). Exemplary ROI-based analysis of signal intensities of the peroneal and tibial portion of the sciatic nerve, the normal appearing musculature, and background noise in patient 5 (a), patient 1 (b) and a healthy control (c). In (a), a distinctive T2-signal increase of the peroneal and tibial nerve in patient 5, and in (b) a T2-signal increase of peroneal division of the sciatic nerve in patient 1 are shown. In the healthy control no pathological nerve signal increase is present (c). In (d) the corresponding SR of the peroneal and tibial portion of the sciatic nerve are shown. In the healthy control, and in the normal appearing tibial portion of the sciatic nerve in patient 1 SR are below 1.7. A SR above 1.7 indicates a lesion of the peroneal portion of the sciatic nerve in patient 1, and in the peroneal and tibial portion of the sciatic nerve in patient 2 (d). **Abbreviations:** p: peroneal portion of the sciatic nerve or peroneal nerve; SR: nerve-to-muscle T2- signal intensity ratio; t: tibial portion of the sciatic nerve or tibial nerve.

The evaluation of MRN was performed in consensus by two neuroradiologists (MW, MP) with more than 4 and 7 years, respectively, of training in MRN. Both raters were unblinded because evidence of any HRS implant was inherent to the evaluated images. The sciatic nerve was evaluated for continuity, alterations in caliber and T2-signal intensity within the tibial and peroneal division. The denervation pattern of proximal and distal target muscles of the sciatic nerve at thigh and calf level was analysed by evaluating increased muscular T2- signal. Using the OsiriX Imaging software [18], a Region of Interest (ROI)- based analysis of the T2- signal intensity of the tibial and peroneal division of the sciatic nerve was performed (Figure 1a, b, c). Nerve-to-muscle signal intensity ratios, abbreviated SR, were determined, to objectify the T2-signal of the nerves. The signal intensity of the nerve was related to the signal intensity of normal appearing musculature not in the distribution of the sciatic nerve, i.e. muscles not innervated by the sciatic nerve (Figure 1d).

## Results

Imaging findings, including denervation patterns of target muscles and the nerve-to-muscle signal intensity ratios (SR) are summarized for all 9 patients and described under one of the following three diagnostic categories relevant to therapeutic management. 1) In two of nine patients imaging evidence of direct mechanical compromise could be raised as an important finding prompting early surgical intervention. 2) In seven of nine patients, sciatic nerve, plexus and root continuity could be documented, ruling out true neuroma formation. 3) In all nine cases T2 signal increase of nerve lesion and denervated muscles could be recorded and objectified to approximate the nerve injury to the pertrochanteric region.

### 1) Constriction of the Sciatic Nerve by Surgical Material in 2 of 9 Patients

In 2 of the 9 cases (patient 1 and 2), MRN revealed constriction of the sciatic nerve by surgical material. In one of these cases (patient 1) resulting in surgical exploration and release of the sciatic nerve from constriction by a cerclage.

**Figure 2:**
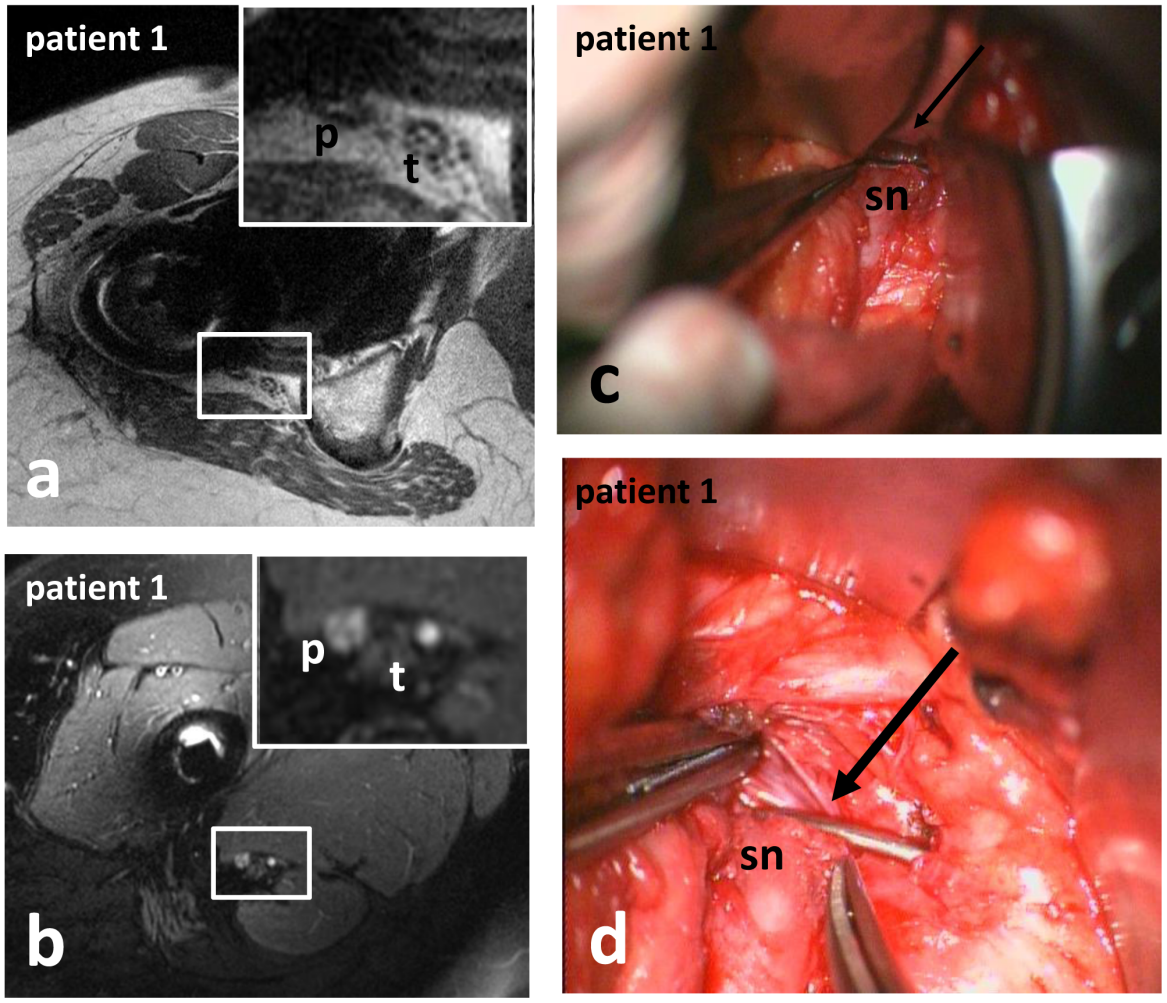
Constriction of the sciatic nerve by surgical material in patient 1 (a, b: siMRN; c, d: intraoperative photographs). T1-weighted sequences, being less vulnerable to susceptibility artifacts, revealed constriction of the peroneal portion of the subtrochanteric sciatic nerve by a cerclage (a). Below the constriction, the peroneal portion of the sciatic nerve exhibited a pathological T2-signal-increase (b). The constriction of the sciatic nerve by a cerclage (arrow in c, d) was validated intraoperatively. **Abbreviations:** p: peroneal portion of the sciatic nerve or peroneal nerve; sn: sciatic nerve; t: tibial portion of the sciatic nerve or tibial nerve.

Patient 1: A 56 year-old female suffered from right sided plegia of the extensors of the foot and toes (MRC muscle strength grade 0/5), and a mild paresis of the flexors of the foot (MRC muscle strength grade 4/5) immediately following ipsilateral HRS 4 months ago (Table 1). MRN revealed constriction of the peroneal portion of the subtrochanteric sciatic nerve by a cerclage (Figure 2a). Consequently surgical exploration of the right sciatic nerve was performed. The constriction of the peroneal portion of the sciatic nerve by a cerclage, resulting in a depression and partial cut of the nerve, surrounded by extensive interfascicular scar tissue (Figure 2c, d) was validated. The peroneal division of the sciatic nerve was released by removal of the cerclage, and the neuroma was excised, because of severe scarring. Direct adaption without tension was not possible, thus interposition of a sural nerve graft was necessary.

**Figure 3:**
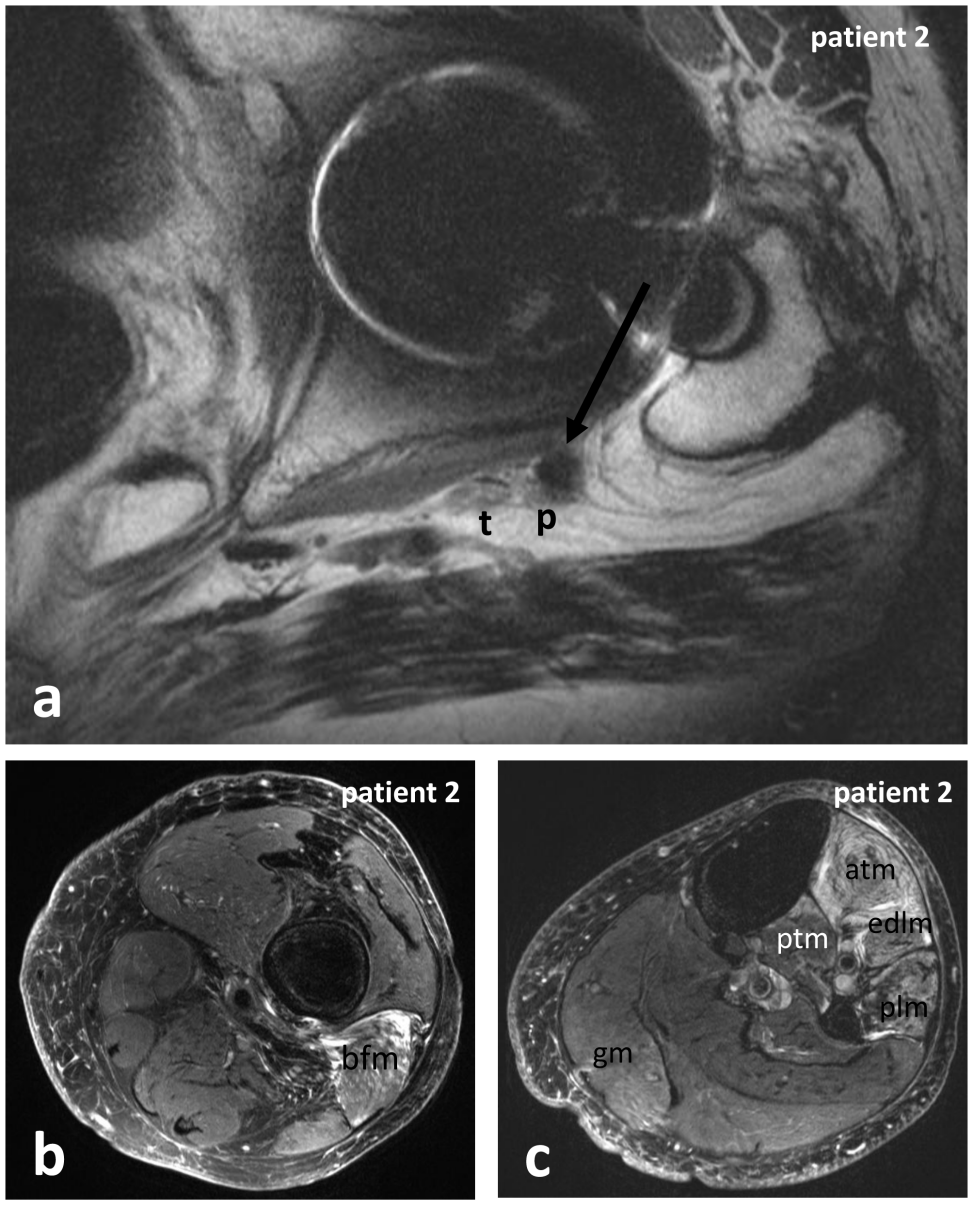
Constriction of the sciatic nerve by surgical material, and denervation of target muscles in patient 2. siMRN revealed compression of the peroneal portion of the left sciatic nerve by a small susceptibility prone foreign body (arrow in a). Besides denervation of the peroneally innervated muscles at the lower leg (c), denervation of the long head of the biceps femoris muscle (b) and slight denervation of the posterior tibialis muscle and the gastrocnemius muscle (c) indicated accompanying affection of the tibial nerve as well. **Abbreviations:** atm: anterior tibialis muscle; bfm: biceps femoris muscle; edlm: extensor digitorum longus muscle; gm: gastrocnemius muscle; p: peroneal portion of the sciatic nerve or peroneal nerve; plm: peroneus longus muscle; ptm: posterior tibialis muscle; sn: sciatic nerve; t: tibial portion of the sciatic nerve or tibial nerve.

Patient 2: A 78 year-old male, suffered from severe paresis of the extensors of the left foot and toes (MRC muscle strength grade 1/5), immediately following an ipsilateral HRS 24 months ago (Table 1). MRN revealed compression of the peroneal portion of the left sciatic nerve by a small susceptibility prone foreign body (Figure 3a). Besides signs of denervation of the peroneally innervated muscles at the lower leg (Figure 3c), signs of denervation of the long head of the biceps femoris muscle (Figure 3b) and slightly of the tibialis posterior muscle and the gastrocnemius muscle (Figure 3c) indicated accompanying affection of the tibial division of the sciatic nerve as well. Since recovery of the sciatic nerve was unlikely to occur two years after HRS, surgery was not indicated.

### 2) Continuity of Sciatic Nerve, Lumbosacral Plexus, and Nerve Roots

**Figure 4:**
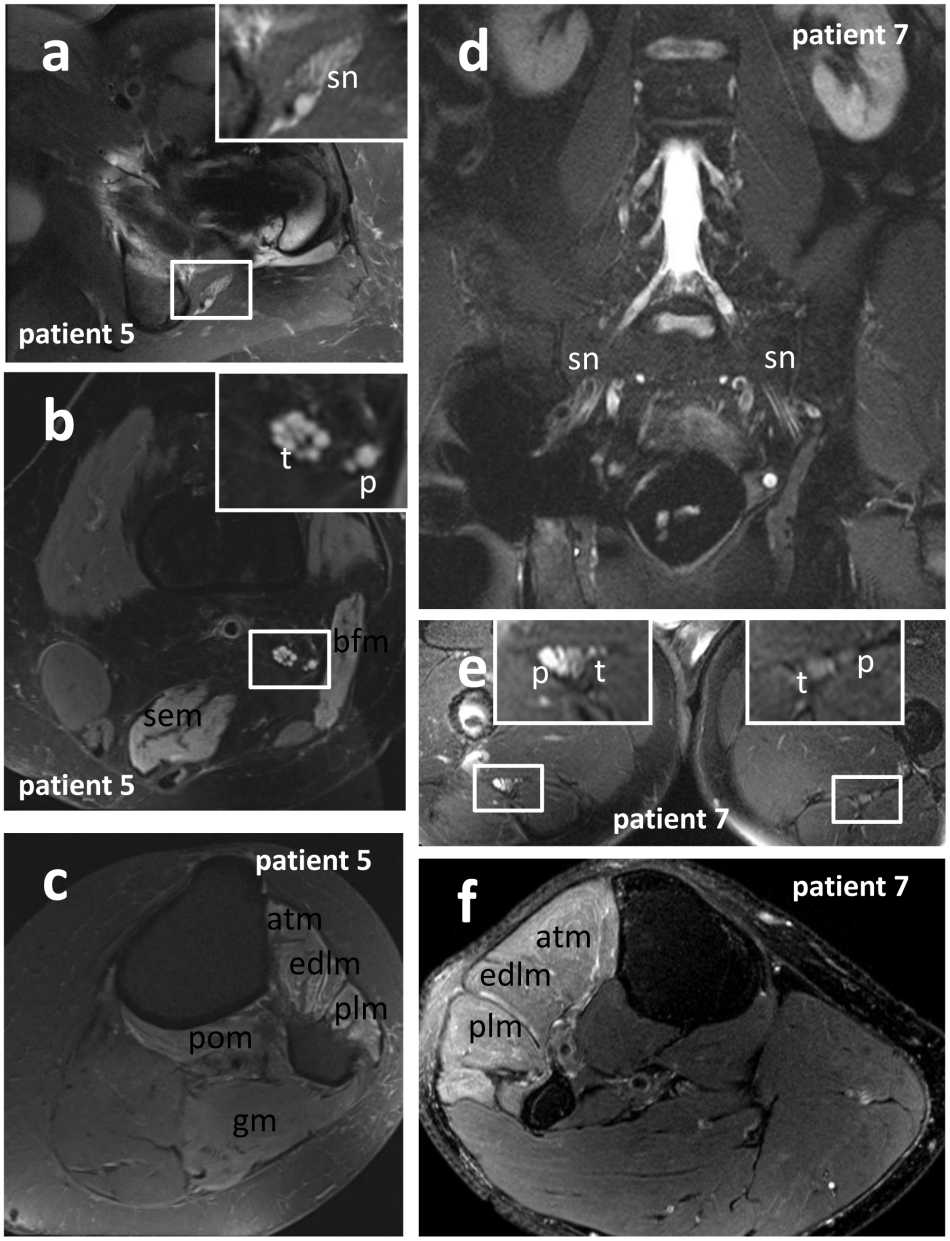
Localization and fascicular distribution of T2-nerve lesion, and denervation pattern of target muscles. siMRN in patient 5 (a, b, c: transversally orientated T2-weighted TSE with fat-suppression) and patient 7 (d: coronally orientated T2 STIR; e, f: transversally orientated T2-weighted TSE with fat-suppression). Depending on the extent of implant-related artifacts, evaluation of the sciatic nerve may be impaired or even impossible for a certain region (d). Regular depiction of the nerve roots and lumbar pexus in patient 7 (d), but the subtrochanteric sciatic nerve exhibits a peroneally accentuated T2-lesion (e), and the peroneally innervated muscles of the proximal lower leg show signs of denervation (f). In patient 5 the proximal sciatic nerve showed a T2-lesion in proximity of the ischial tuberosity (a), affecting all fascicles of the nerve at level of the hip (a) and thigh (b). Furthermore the denervation of the biceps femoris and semimembranosus muscle of the thigh (b), and the anterior tibialis, extensor digitorum, peroneus longus, popliteus and gastrocnemius muscle of the lower leg (c) indicate affection of the tibial and peroneal division of the sciatic nerve. **Abbreviations:** atm: anterior tibialis muscle; bfm: biceps femoris muscle; edlm: extensor digitorum longus muscle; gm: gastrocnemius muscle; p: peroneal portion of the sciatic nerve or peroneal nerve; plm: peroneus longus muscle; pom: popliteus muscle; sem: semimembranosus muscle; sn: sciatic nerve; t: tibial portion of the sciatic nerve or tibial nerve.

In the 7 of 9 patients in whom a proximal lesion of the lumbosacral plexus could not be ruled out via clinical examination and electrophysiology, MRN could eventually document that the lumbar nerve roots and the lumbar plexus proximal to the implant related artifacts were intact (Figure 4d), without spinal or intraforaminal compression of the nerves. The extent of artifacts varied between different patients. In general, the artifacts reached their greatest extent in the pertrochanteric region, where the diameters of the implants were maximal (Figure 4a, d). Susceptibility related signal loss was less extended in the T1-weighted sequences than in the fat-suppressed T2-weighted sequences, owing to the short echo time (TE) of T1-weighted TSE. Thus on T1-weighted TSE sequences implant related artifacts were minimal, and most parts of the sciatic nerve were evaluable, so that continuity of the sciatic nerve could be proven with sufficient diagnostic confidence.

### 3) Lesion Localization by Evaluation of Nerve T2-signal Intensity, Fascicular Lesion Distribution, and Denervation of Target Muscles

In 7 of 9 patients the nerve T2-lesion could not be visualized directly due to implant-related pertrochanteric artifacts, but it was possible to approximate the level of the lesion indirectly by evaluating the sciatic nerve proximally and distally to the artifacts (Figure 4). In fat suppressed T2-weighted TSE sequences, hyperintense signal alterations exceeded the area of signal loss up to 4 cm. Thus, even when continuity and caliber of the sciatic nerve were depicted regularly on T1-weighted sequences, the nerve T2-signal in the proximity of the implant was not evaluable until the T2-hyperintense signal alterations of the surrounding ceased to normal. Distal to the artifacts, the T2-signal intensity of the tibial and peroneal division of the sciatic nerve was measured (Figure 1a, b) and related to the signal intensity of normal appearing muscles not innervated by the sciatic nerve (Figure 1d). Clinically affected nerves regularly exhibited a signal intensity ratio (SR) above 1.7, ranging from 1.8 to 4.1, over at least 4 cm length (Table 1). The corresponding SR in both legs of 10 healthy individuals ranged from 1.1 to 1.7 (mean value 1.4). In all 9 patients the tibial and/or peroneal division of the subtrochanteric sciatic nerve showed pathological increase of its intrinsic T2-signal and of the SR above 1.7, over at least 4 cm distance (Table 1). The differences in averaged SR between clinically affected patients and healthy controls were significant (two sample T-test: p<0.001) at a cut off value of 1.7 of normalized T2-signal. Corresponding to the clinical findings, T2-lesions of affected sciatic nerves showed a predilection for the peroneal portion (Table 1). The denervation pattern of the target muscles of the sciatic nerve at thigh level was determined in all 9 patients (Table 1). The short head of the biceps femoris muscle is innervated by the peroneal division of the sciatic nerve; the long head of the biceps femoris muscle, the semimembranosus, semitendinosus, and parts of the adductor magnus muscle are innervated by the tibial division of the sciatic nerve. The denervation pattern of the muscles innervated by the peroneal nerve at calf level was determined in 7 of 9 patients (Table 1), as two patients (patient 1 and patient 4) aborted the MRN examination before completion. The anterior tibialis, peroneus, extensor hallucis, and extensor digitorum muscles are innervated by the peroneal nerve. The tibial nerve innervates the gastrocnemius, soleus, popliteus, plantaris, posterior tibialis, flexor digitorum brevis and longus muscle. In all of these 7 patients, the pathologic signal increase of the tibial and/or peroneal portion of the sciatic nerve was associated with signs of denervation of at least one target muscle at calf level (Table 1). Denervation at thigh level was detected in 5 of 9 patients, indicating a proximal lesion of the sciatic nerve as well.

### Correlation of Nerve Lesions Diagnosed by MRN and Decreased Muscle Strength of Target Muscles in Clinical Examination

In every patient the muscle strength of the target muscles of the peroneal and tibial nerve was tested and classified according to the scale established by the Medical Research Council (MRC) (Table 1). For every patient, clinical muscle strength of target muscles was compared to SR of the innervating peroneal and tibial nerve (with MRC muscle strength equal or smaller than 4/5 indicating weakness of target muscles, and a SR above 1.7 indicating a lesion of the innervating nerve). Thus comparing SR of the peroneal and tibial nerve in all nine patients, a total of 18 nerves was tested. In 13 of 18 muscle strength and SR were pathologic, in 2 of 18 muscle strength and SR were normal, in 1 of 18 muscle strength was pathologic but SR was normal, and in 2 of 18 muscle strength was normal but SR was pathologically elevated. Thus, compared to clinical testing of the muscle strength grades of target muscles, siMRN with determining the SR had a sensitivity of 0.93 (95% CI 0,685 to 0,987), a specifity of 0.50 (95% CI 0,15 to 0,85), a positive predictive value of 0,867 (95% CI 0,621 to 0,963), and a negative predictive value of 0,667 (95% CI 0,208 to 0,939).

These results indicate that, by evaluating the signal intensity of the peroneal and tibial portion of the sciatic nerve, and the denervation pattern of target muscles, the location of the nerve injury could be localized to the pertrochanteric level, and correlated reliably with clinical diagnosis of affection of the tibial and/or peroneal nerve.

## Discussion

### Diagnostic Testing in Sciatic Nerve Injury

Neurological examination combined with nerve-conduction-studies and EMG is the gold standard in the evaluation of peripheral nerve injuries. Recently MRN has emerged as an additional diagnostic method for the evaluation of peripheral nerve lesions [16], [19]–[25]. MRN is capable to display the peripheral nervous system from the nerve roots, along the plexus to distally located peripheral nerves. With transverse imaging sections, MRN at a magnetic field strength of 3 Tesla is capable of visualizing not only nerve trunks but also the nerve fascicles. In sciatic neuropathy after HRS it is diagnostically most important to determine lesion localization precisely and to rule out or suggest severe mechanical compromise, which would prompt early intervention with the aim of surgical nerve release. Both for proximal lesion determination at pertrochanteric level and visualization of mechanical nerve compromise, as aforementioned, the diagnostic imaging modalities usually employed may have difficulties. Although MRN is the method of choice for visualizing nervous structures, in the presence of susceptibility artifacts, adjacent to metallic objects, such as after HRS, its diagnostic value has not been challenged so far. In this study we therefore employed MRN optimized for reducing susceptibility related artifacts in the proximity of metallic implants. With this method we were able to diagnose constriction of the sciatic nerve by surgical material in 2 of 9 patients. Using T1-weighted TSE sequences, being less vulnerable to susceptibility artifacts than fat-suppressed T2-weighted sequences, continuity of the sciatic nerve of the remaining 7 patients could be proven with sufficient diagnostic confidence. Although the exact localization of the T2-lesion could not be visualized directly in these 7 patients on fat-suppressed T2-weighted TSE sequences due to implant related artifacts, it could be approximated to the pertrochanteric region in all these patients, by evaluation of the regions proximal and distal to the artifacts.

### MRN Findings in Sciatic Neuropathy Related to Hip Arthroplasty

In fat-saturated T2-weighted MRN, peripheral nerves show intrinsic, hyperintense signal alterations at the location of a lesion, and in severe axonal lesions after a certain period of time often distal to the lesion as well, indicating Wallerian degeneration [26]. In chronic entrapment lesions, additional increase of nerve caliber indicates severe neuropathy [19]. Approximately ten days after denervation, the innervated muscles distal to the lesion show spontaneous activity on electromyography (EMG). An increased signal on T2-weighted images is found after 48 hours [27]. During regeneration T2-signal increase of nerve and muscles regress and in parallel with pathological spontaneous activity as well decline.

Although, even with siMRN a certain short segment of the sciatic nerve may not be evaluable because of metal- related susceptibility artifacts, we were able to diagnose 1) direct mechanical compromise of the nerve by surgical material in two of nine patients. In one of these two patients the indication for surgical exploration could be based on MRN. The sciatic nerve was released by removal of a cerclage, and a sural nerve graft had to be interposed. In the second case, in which direct compromise of the nerve through surgical material was diagnosed by MRN, surgery was not performed, because recovery of the sciatic nerve was unlikely to occur two years after HRS. 2) The continuity of nerve roots, plexus, and proximal sciatic nerve, was confirmed in seven of nine patients, ruling out true neuroma formation. 3) The level and fascicular distribution of the nerve lesion could be indicated reliably in all nine patients. Even if large segments of the nerve, including the site of the lesion, are hidden by susceptibility artifacts, the intrinsic T2- signal intensity of the nerve proximal and distal to the artifacts, and signs of denervation of the target muscles can be determined reliably by siMRN. These findings allow to deduce the level and extent of the lesion of the innervating nerve or nerves. In all 9 patients the tibial and/or peroneal division of the subtrochanteric sciatic nerve showed pathological increase of its intrinsic T2-signal and of the nerve-to-muscle T2- signal intensity ratio (SR) above 1.7, over at least a distance of 4 cm (Table 1). Thus, in HRS related injury there seems to exist a cut- off value of SR, differentiating healthy sciatic nerves from HRS related sciatic nerve injury despite the difficulty of implant related artifacts. Of course this cut- off value may vary between different pulse sequences, but with the T2- weighted SE- sequence used in this study, this cut- off was determined at a value of 1.7 of SR. Hence, despite expected susceptibility artifacts, siMRN in addition to electrophysiological testing, may indicate the site and fascicular distribution of a nerve lesion and is consequently a useful diagnostic method before surgical exploration.

### Reduction of Artifacts from Prosthetic Implants in MR Imaging

Certain methods for optimizing artifacts adjacent to orthopedic implants in general and hip prosthesis in particular have been described [28]–[31]. Factors influencing susceptibility artifacts adjacent to metallic implants include size and composition of the implant, orientation of the implant with regard to the static magnetic field (B0), strength of B0, type and parameters of the pulse sequence, and other imaging parameters like bandwidth, echo train length and voxel size [29]. The metallic composition of the implant has a major influence on the extent of the susceptibility artifacts, with non-ferromagnetic titanium alloy producing much less artifacts than ferromagnetic stainless steel. As a matter of cause, the size of the implant influences the extent of the artifacts, with small implants producing lesser artifacts than large ones. The position of the patient und thus of the implant within B0 should be considered as well. If possible, the long axis of the implant and the direction of the static magnetic field should be parallel, since susceptibility artifacts augment with increasing angle of the long axis of the implant from the direction of B0. The choice of an adequate pulse sequence is important. Instead of gradient echo (GRE) sequences, spin echo (SE) or turbo spin echo (TSE) sequences should be used. The 180° refocusing pulse applied in SE sequences corrects for large magnetic field inhomogeneities, leading to less dephasing artifacts. However, even SE or TSE sequences are still sensitive to other types of metal-induced artifacts as pile-up artifacts in readout- and slice direction [32]. Fat-saturation is practically mandatory in T2-weighted MRN. With spectral or frequency-selective fat-saturation, being very frail for magnetic field inhomogeneities, short inversion recovery (STIR) sequences for fat-saturation should be used instead, being less dependent on the homogeneity of the magnetic field. Higher magnetic field strengths produce larger susceptibility artifacts. But with higher gradient strength used in high field MRI, the increased distortion effects of higher static magnetic fields can be reduced by consequently applying of higher gradient strengths, allowing higher readout bandwidth at a given field-of-view. Thus optimizing image acquisition parameters produces comparable susceptibility artifacts at 1,5 and 3 Tesla, and the optimal image quality at 3 Tesla may be qualitatively superior compared to that at 1,5 Tesla [30]. Field-of-view, image matrix, and section thickness determine voxel size. Small voxel size increases spatial resolution, and has little or no effects on artifacts. Using a small field-of-view, high resolution matrix, thin sections, and high gradient strengths reduce susceptibility artifacts. The echo train is defined as the set of echoes that follows the application of a single excitation pulse. The echo train duration is the period during which these echoes are acquired. The number of echoes in the echo train is the echo train length. With a constant echo train duration assumed, increasing the echo train length reduces susceptibility artifacts. Choosing an adequate pulse sequence and considering the aforementioned variable parameters, we refer to MRN optimized for reduction of metal-related image artifacts as susceptibility insensitive (si)MRN. Although all examinations were performed with an MRI scanner with 3T field strength, potentially producing more susceptibility artifacts than scanners with lower field strengths, image quality may benefit from the relatively high gradient pulses.

### Higher Vulnerability of Peroneal Compared to Tibial Division of Sciatic Nerve

Regardless of the mechanism of injury, the peroneal division of the sciatic nerve is more vulnerable to traumatic damage, and its potential for recovery is restricted compared to the tibial division of the nerve [2], [3], [33]. Consequently, the patients with sciatic nerve palsy after HRS that were included in our study clinically showed a peroneally accentuated lesion of the affected sciatic nerve (**Table 1**). This predilection to injury of the peroneal division of the sciatic nerve was confirmed by siMRN, showing a significant increase in the ratio of signal intensity of the nerve compared to normal appearing muscle, and a peroneally accentuated denervation pattern of target muscles (**Table 1**). The lateral localization of the peroneal division of the sciatic nerve puts it at risk for injury, but this vulnerability may be related to reasons other than mere anatomical localization [2].

### Treatment of Sciatic Nerve Palsy Related to Hip Arthroplasty

As with most peripheral nerve injuries, there is no generally accepted consensus and no commonly accepted guideline to be consulted for optimal management of injuries of the sciatic nerve related to HRS. Generally there is consensus, that surgical intervention is only indicated for severe nerve lesions without sufficient potential for spontaneous recovery. Of course sharp transections of the nerve should be repaired as soon as possible, at least within 72 hours to prevent retraction of the nerve [3]. When compression or constriction of the nerve by surgical material is the verifiable cause of the deficit, indication for surgical exploration and release of the nerve is unequivocal and the operation should be performed as soon as possible. When the nerve has been injured by stretching or contusion, there is no general consensus on the optimal period of waiting for the nerve to recover, before surgical exploration is considered. Experienced surgeons recommend surgical exploration for severe neurologic deficits that do not show spontaneous recovery over a few months. A waiting period of three months for the nerve to recover clinically and electrophysiologically is widely accepted, because when clinical or electrophysiologic regeneration occurs, most patients show subsequent recovery to acceptable function under conservative treatment [2], [3], [7], [33]. In one of the patients presented in this study, who suffered from sciatic nerve palsy after HRS, constriction of the peroneal division of the sciatic nerve by a cerclage was diagnosed via siMRN (**Figure 2a**). Consequently surgical exploration and neurolysis were performed, to confirm this suspicion. A cerclage constricted and partially dissected the peroneal division of the sciatic nerve (**Figure 2c, d**) and was consequently removed.

## Conclusion

In addition to the gold standard of thorough physical and electrophysiologic testing, siMRN is a valuable diagnostic tool in patients with sciatic nerve palsy related to HRS, especially when surgical exploration of the nerve is considered. Despite implant-related artifacts, siMRN with optimized pulse sequences helps reducing artifacts and increases diagnostic value, capable to visualize mechanical nerve compromise. Furthermore siMRN may reveal typical patterns of denervation, and potentially the fascicular distribution of the lesion. Due to remaining susceptibility artifacts, the exact location of the lesion is often not visualized directly, but can be approximately deduced by evaluation of the nerve proximal and distal to the artifacts. Of course, siMRN cannot completely eliminate, but only reduce metal-related artifacts to a certain extent. Thus further improvement of this method may lead to minimize the remaining limitations and further increase its diagnostic value.
